# Variable effects of vegetation characteristics on a recreation service depending on natural and social environment

**DOI:** 10.1038/s41598-023-27799-7

**Published:** 2023-01-13

**Authors:** Masahiro Aiba, Rei Shibata, Michio Oguro, Tohru Nakashizuka

**Affiliations:** 1 Research Institute for Humanity and Nature, 457-4 Motoyama, Kamigamo, Kita-ku, Kyoto, 603-8047 Japan; 2 Faculty of Agriculture, Niigata University, Niigata, 950-2181 Japan; 3 Forestry and Forest Products Research Institute, Tsukuba, 305-8687 Japan

**Keywords:** Ecosystem services, Forest ecology

## Abstract

In this study, we examined roles of three vegetation characteristics in provisioning of a recreation service by applying a machine-learning method to 4,708,229 spatially-explicit records of hiking activity in Japan. Then, expected impacts of land-use changes assessed and mapped based on the model. Associations between a recreation service and three vegetation characteristics were considerably variable depending on the social and natural environment such as accessibility and altitude. As a consequence, expected impacts of unit changes in vegetation characteristics on the service flow were considerably heterogeneous throughout the study area. The signs (positive or negative) of the impact can be reversed depending on the contexts even among nearby sites. Such notable but variable contributions of vegetation on a recreation service should be carefully reflected in landscape management. Even moderate changes in either the quantity or quality of vegetation can have a considerable impact on the frequency of hiking activity. Landscape management for promotion of the recreation service should be carefully designed for each locality on the grounds of the context-dependent effects of vegetation.

## Introduction

Understanding how various landscape characteristics including ecological, environmental, and social attributes influence an ecosystem service is essentially important for assessment, planning, and management for the sustainable enjoyment^1–4^. Especially, identification of the relationships between ecological structures (e.g. species, communities, and vegetation characteristics) and ecosystem services is crucial to assess impacts of ecosystem changes on the services. This is because, without such knowledge, human impacts on the ecological structures (e.g. extinctions of species or vegetation shift due to climate change) cannot be fully reflected in an assessment of ecosystem services^5^. For example, empirical knowledge on wild bee’s dependence on some types of land-use available as nesting sites enabled to build an estimation model for crop pollination service^6^. The model was implemented in InVEST^7^ and has been used in numerous studies assessing impacts of land-use changes on the service^8,9^.

However, for cultural ecosystem services (CES), most of the assessments and forecasts have been performed without basis of knowledge of how ecological structures contribute to a service^10^. A common strategy for spatial assessments of CES is a proxy-based method that assigns a single value of a service to each land cover class based on an evidence collected often elsewhere outside of the study area^3,11^. Although the low reliability of the method that does not reflect ecological heterogeneity within a land cover class has been criticized, the method is still very popular possibly because, for CES, both collection of a primary data and development of a model that explicitly associates a service with ecological structures are difficult.

Recently, some empirical models have been developed to understand associations between ecological structures and CES^12–17^. These studies have revealed that CES can vary with ecological structures at least in some cases. For example, Ridding et al*.*^13^ demonstrated that proportion of forest positively associates with people’s preferences as a place of outdoor recreation. However, these models are often relatively simple and/or assume a consistent effect of ecological structures throughout a study area. This is possibly because modeling of CES often requires a relatively large number of explanatory variables encompassing ecological, environmental, and social factors, and therefore making the model further complex is not easy. However, there are plenty of evidences that an association between ecological structures and a CES, whose demand depends on sociocultural backgrounds of beneficiaries, is complex and context-dependent. For example, Termansen et al*.*^16^, who analyzed a forest recreation service in Denmark, found that people in areas characterized by higher average income enjoyed forest recreation more frequently. For another instance, Schirpke et al*.*^14^ showed that perception of Alpine landscapes is significantly different between tourists and residents.

If such a context-dependence is important for associations between a CES and ecological structures, spatial assessments of the services based on an average effect portrayed by a simple model (e.g. unit increase in natural vegetation uniformly promote a service flow throughout a study area irrespective of contexts) will be essentially misleading. One of the main purposes of a spatial assessment of an ecosystem service is to quantify spatial heterogeneity in effects of an artificial disturbance or a management policy on ecosystem services. Not only spatial heterogeneity in artificial disturbances but also heterogeneous responses of a service to unit change in ecological structures would be responsible for spatial heterogeneity in a CES. Therefore use of an over-simplified model that ignored heterogeneity in the association would skew the result. However, a relative impact of such heterogeneous associations in spatial assessments of a CES has been rarely examined.

Modeling the associations by using some types of machine-learning methods can be an effective solution of this problem. Given sufficiently large dataset, machine-learning technique such as boosted regression trees (BRT) enables efficiently building a flexible model where high-dimensional interactions and non-linear effects of numerous variables are properly accounted. Although previous studies that employed machine-learning to model a CES have focused only on average effects of the explanatory variables^12,18^, such flexibility of the technique is ideal for exploration of context-dependent contributions of ecological structures to a CES provisioning.

In this study, we applied BRT to 4,708,229 spatially-explicit records of hiking activity in Japan, which were obtained from a social networking service for hikers as a proxy of a recreation service flow. This dataset has advantage in interpretability of relationships between ecological structures and CES over data from general-purpose social media (e.g. Flickr and Instagram) because all users of the service receive CES via a single activity, hiking. Number of records for 4244 approximately 10 km grids was modeled as a function of 50 ecological, environmental, social and infrastructural variables. Then extent of context-dependence in association between the recreation service and three vegetation variables (total vegetation cover, proportion of natural vegetation to total vegetation, and proportion of primary vegetation to natural vegetation) was examined. Specific questions are (i) To what extent association between a recreation service and vegetation variables are context-dependent? (ii) What contexts are responsible for heterogeneity in association between the service and the vegetation variables?, and (iii) What is the importance of such context-dependent association for sustainable management of the service?

## Results

**Figure 1:**
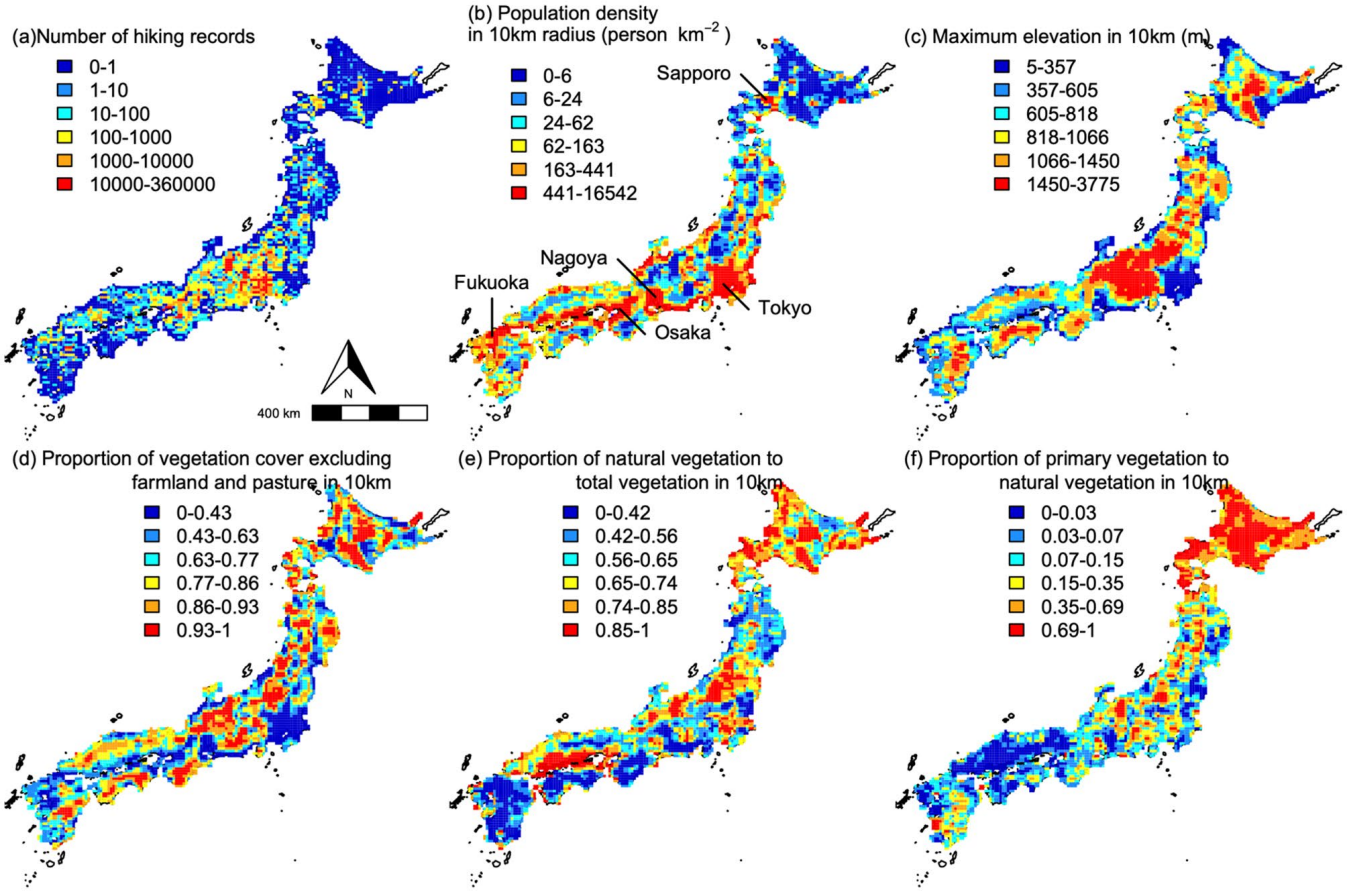
Maps of important variables in the study area summarized for the standard grids. (**a**) Number of hiking records, (**b**) population density in 10 km radius from the center of each grid with some big cities, (**c**) maximum altitude in 10 km, (**d**) total vegetation cover in 10 km, (**e**) proportion of natural vegetation to total vegetation cover in 10 km, (**f**) proportion of primary vegetation to natural vegetation in 10 km. The maps were drawn using R 4.1.1 software package^29^ with public data of the border (https://www.gsi.go.jp/kankyochiri/gm_jpn.html).

**Figure 2:**
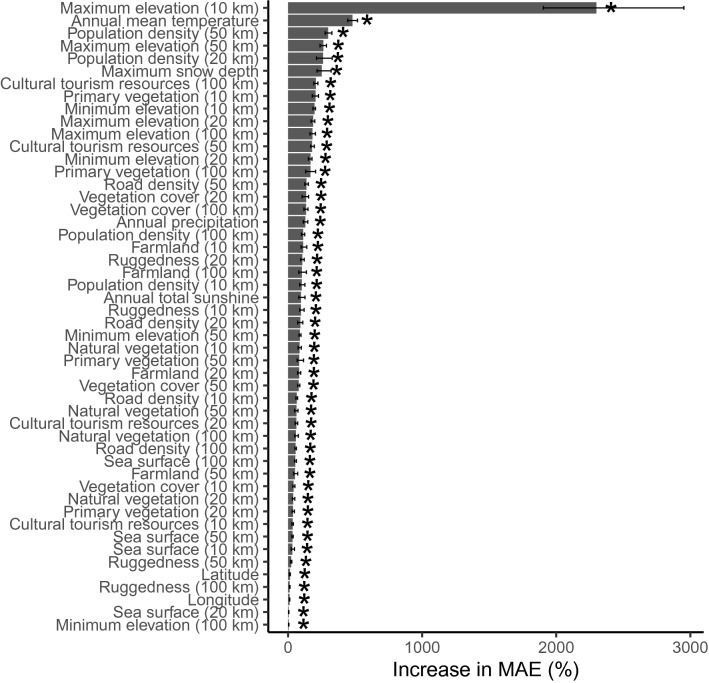
Importance of explanatory variables for total number of hiking records. The bar indicates 95% interval after 100 permutations. Values with an asterisk are statistically significant at p < 0.05. The values in parentheses are the spatial scale (distance from the center of each grid, radius).

Spatial patterns of the essential variables were summarized in Fig. 1. Thirty six percent of the variance of the number of hiking records were explained by our model (i.e. cross-validated R^2^ was 0.36). All of the fifty ecological, environmental, and social/infrastructural variables were significant at P < 0.05 (Fig. 2). Among the variables, importance of maximum elevation in 10 km was dominant and followed by climate variables such as annual mean temperature and maximum snow depth and population density in multiple scales (Fig. 2). In terms of the absolute size of effects, importance of vegetation variables (i.e. total vegetation cover, proportion of natural vegetation to vegetation cover, proportion of primary vegetation to natural vegetation) were also not negligible. For example, mean absolute error increased 305% and 235% after permutation of proportion of primary vegetation in 10 km radius and total vegetation cover in 20 km radius, respectively. Although spatial scales at which each vegetation variables are most important were variable, hereafter we focus on variables at the scale of 10 km radius because land-use changes at the larger scales are unusual in Japan.

**Figure 3:**
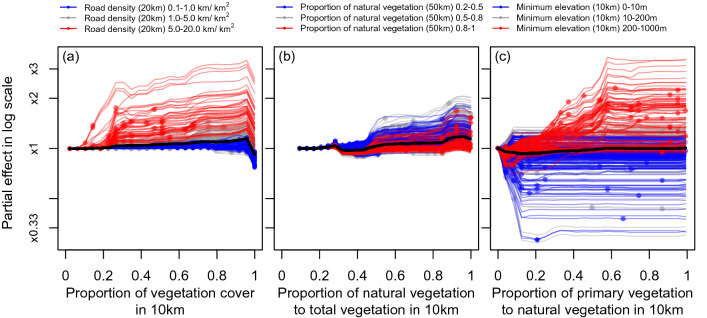
Individual conditional expectation plots for (**a**) Total vegetation cover in 10 km, (**b**) Proportion of natural vegetation to total vegetation cover in 10 km, (**c**) Proportion of primary vegetation to natural vegetation in 10 km. Partial effects of each explanatory variable were shown in log scale. Expected responses for each grid were centered to be zero at the low end of an explanatory variable. The value of a variable whose interaction with a vegetation variable was important was shown by color of a line. Filled circles indicate observed values of a vegetation variable for each site. A black line indicates an average effect. Five hundred sites were randomly sampled for visualization to avoid the plots being too dense.

On average, associations of the record number with vegetation variables at the scale of 10 km radius seem weak (Fig. 3, black lines). However, the average trends are misleading because the estimated associations were quite variable among sites. For example, record number increased fourfolds or decreased by one-fourth with increasing proportion of primary vegetation from 0 to 1 depending on sites (Fig. 3c).

**Figure 4:**
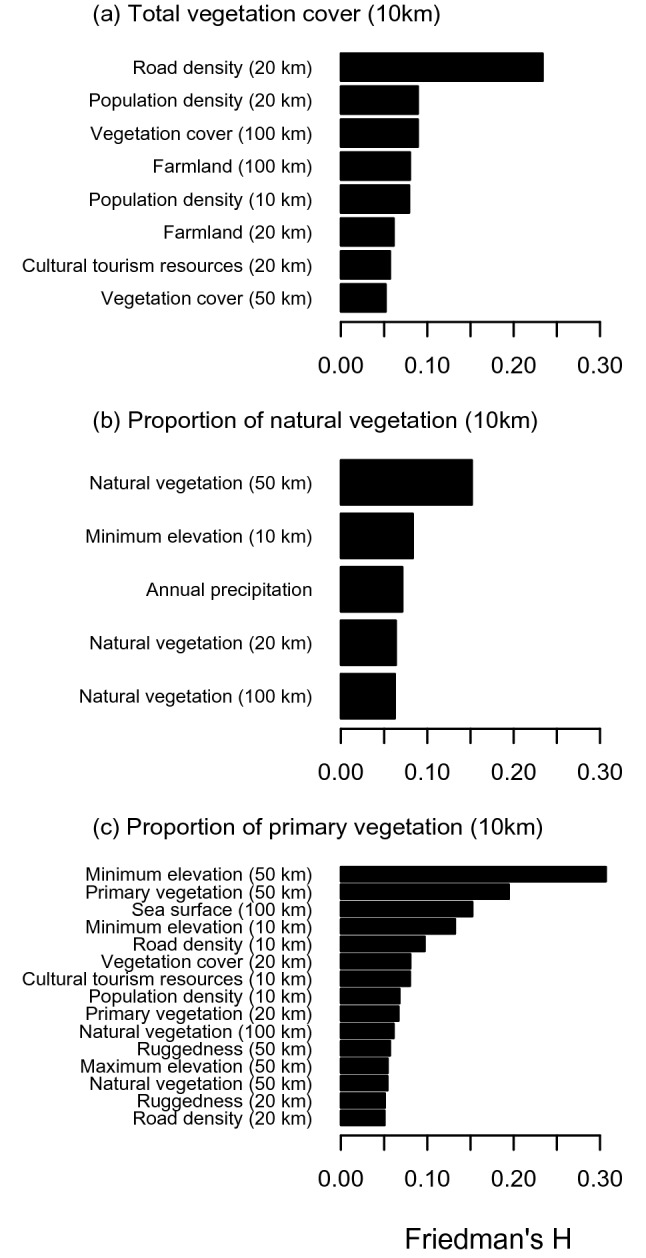
Relative importance of interactive effects evaluated by Friedman’s H statistic for each of the three vegetation variables. Only variables with Friedman’s H ≥ 0.05 were shown. The values in parentheses are the spatial scale (distance from the center of each grid, radius).

Friedman’s H values, which indicate importance of interactive effects as a proportion between 0 and 1, were relatively high for some variables, indicating that interactions with these variables are responsible for the heterogeneous associations (Fig. 4). The most important interactions were that with road density in 20 km for total vegetation cover, that with proportion of natural vegetation in 50 km for proportions of natural vegetation, and minimum elevation in 10 km for proportions of primary vegetation.

While associations with vegetation cover were consistently weak in less-accessible (road density in 20 km radius < 1 km km^−2^) sites but it was often considerably positive in more-accessible (road density in 20 km radius ≥ 5 km km^−2^) sites (Fig. 3a). Positive association with proportion of natural vegetation in 10 km was weaker in sites with higher proportion of natural vegetation at a regional (50 km) scale (Fig. 3b). Associations with proportion of primary vegetation were often positive in high-altitude sites (minimum elevation in 10 km ≥ 200 m) but turned to negative in low-altitude sites (minimum elevation in 10 km < 10 m, Fig. 3c).

**Figure 5:**
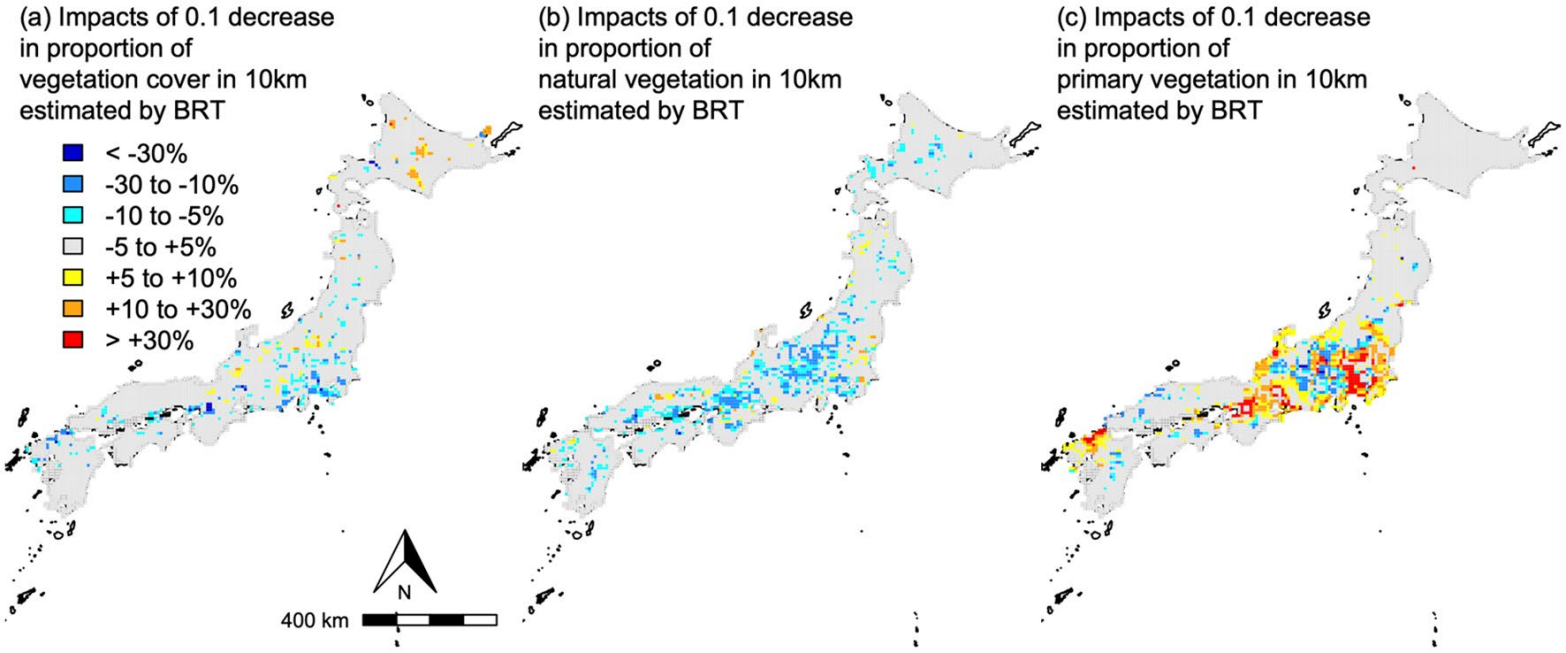
Expected impacts of 0.1 decrease in the three vegetation variables on total number of hiking records. (**a**) Impacts of loss in vegetation cover, (**b**) proportion of natural vegetation, and (**c**) proportion of primary vegetation. For maps of each month, see Figs. S1–3. The maps were drawn using R 4.1.1 software package^29^ with public data of the border (https://www.gsi.go.jp/kankyochiri/gm_jpn.html).

As a result of these context-dependent associations as well as the nonlinear, and sometimes non-monotonic, associations in the BRT model, expected responses of the recreation service flow to unit (0.1) decrease in vegetation variables were considerably heterogeneous throughout the study area (Fig. 5a–c). For all the three vegetation variables, the 0.1 decreases caused more than ± 30% change in the service flow at least in some sites. Because the variables responsible for the context-dependence, e.g. road density in 20 km, can greatly differ at a scale of tens of kilometers, predicted impacts of vegetation degradation were also variable at the relatively small spatial scale.

## Discussion

Vegetation variables, physical environment and social/infrastructural environment at various spatial scales were all important determinant of number of hiking records, which we considered as a surrogate of a recreation service flow. Importance of interactive effects among these variables sometimes exceeded 20% of the total effect of each variable, indicating that the relationships between these environmental variables and the service can be context-dependent. These results reinforce our previous finding that the recreation service is provisioned through interactions of biotic, abiotic and social environment at multiple spatial scales, which was obtained in the analysis performed on a tiny subset of the data analyzed in this study^12^. Associations between number of hiking records and vegetation characteristics considerably varied depending on contexts such as accessibility and elevation. As a result, the average trends of the associations can be misleading summaries of the associations. For example, on average, number of hiking records appears almost independent of proportion of primary vegetation at the 10 km scale although either positive or negative associations were expected in numerous sites. Hereafter we focus on such context-dependent associations between the recreation service and vegetation characteristics. For general discussions the delivery process of the service, see Aiba et al*.*^12^.

Frequency of hiking activity increased with total vegetation cover only in a highly accessible sites, which are often characterized also by dense population in the surrounding area. In contrast, total vegetation cover was not sufficient for attraction of hikers in less-accessible, sparsely-populated areas. It seems reasonable that urban residents who live in highly developed environment appreciate activities in easily accessible vegetated areas irrespective of the vegetation quality. It seems also reasonable that some difference of vegetation cover in rural area, where vegetation cover is generally high, cannot be a key factor for both local residents and visitors from cities. Although both vegetation cover^13,19,20^ and accessibility^12,13,21^ have been identified as an important factor for CES, to our knowledge this is the first evidence for importance of their interaction. As a result, negative effect of 0.1 decrease in proportion of vegetation cover was expected mainly in suburban area close to densely populated areas such as Tokyo and Osaka throughout a year (see Figs. 5a and S1 with Fig. 1b). In contrast, the expected effects of 0.1 decrease were often positive in some isolated, highly-vegetated (total vegetation cover > 0.9), alpine areas such as the Japanese Alps and the Daisetsuzan mountains especially in winter. This is possibly because high vegetation cover act as a surrogate of inaccessibility of these areas which are not well-captured by other variables in our model e.g. long walking time, absence of public transport, closure of a road in winter^15^.

Positive associations between hiking record number and proportion of natural vegetation in 10 km was rather consistent in regions with low to mid proportions of natural vegetation at a regional (50 km) scale. The association was most remarkable and widespread in spring and autumn (Fig. S2), suggesting that seasonal attractions of natural vegetation, e.g., spring ephemerals, fresh green, and autumn colors, are important for the pattern. Hikers cannot expect these attractions in plantations because plantation species are mostly evergreen conifers. As a result, in spring and autumn, negative impacts of loss of natural vegetation were expected in numerous sites of both suburban and rural areas. In contrast, the positive association was weak in sites where proportion of natural vegetation is very high at a regional (50 km) scale. It is not surprising that proportion of natural vegetation cannot be a key for destination choice in an area where natural vegetation is generally high.

Context dependence was most remarkable for the association between the recreation service and proportion of primary vegetation. Hiking record number increased with increasing proportion of primary vegetation in 10 km in high-altitude sites but decreased with the variable in low-altitude sites. Positive associations (negative impacts of primary vegetation loss) were detected widely in montane area in spring and autumn but in summer they were observed only in alpine areas of central Japan (see Fig. S3 with Fig. 1c). This seasonal pattern suggests that fresh green and autumn colors, which are common features in cool-temperate primary forests, attract hikers in spring and autumn, respectively while alpine meadows in high mountains do so in summer. Although both fresh green and autumn colors are normally found in cool-temperate natural forests, some tree species popular for their beauty (e.g., beech for fresh green and some maple species for autumn color) are characteristic to a primary forest^22^. In the study area, many alpine flower species can be observed mostly in protected primary vegetation on a high mountain.

In contrast, the associations were consistently negative (impacts of primary vegetation loss were positive) throughout a year in low-altitude sites of central Japan. The avoidance of primary vegetation in lowland by hikers may be partly explained by the fact that secondary forests can be deciduous forests even at a site originally covered with warm-temperate evergreen forests^22^. As described above, evergreen forests lack attractions for hikers such as fresh green, spring ephemerals, and autumn colors. In addition, in Japan, many citizens prefer deciduous forests because they consider a deciduous secondary forest is an essential component of a typical, traditional, and culturally important suburb landscape (i.e. *satoyama*)^23,24^. Although such people’s preference for deciduous forests may partly derive from the Japanese culture, similar trends that prefer a deciduous forest to an evergreen forest in a recreation use have been reported also in studies in Western countries^16,25^.

The notable but complex, context-dependent associations between the recreation service and vegetation characteristics should be reflected appropriately in the management of the landscape. Our analysis demonstrated that even moderate modification in either quantity and quality of vegetation at a local scale can have a considerable impact on a frequency of hiking activity, i.e. a recreation service flow. Ill-advised landscape management would generate a negative effect on not only hiker’s well-being but also local economy relying on consumption of visitors. For example, only 0.1 (i.e. 10%) decrease in proportion of natural forest can result in > 10% decline of the service flow (Fig. 5b) in exchange for increased timber production. Another implication of our analysis is that landscape management for promotion of the recreation service should be carefully designed for each locality on the ground of the context-dependent associations with vegetation. For example, a policy to increase any type of vegetation will be effective in a suburban area while strict protection of primary vegetation and conversion of plantation to natural forest will be more successful in a rural area.

Although it is difficult to obtain these insights without using a huge data accumulated on a social media, care should be taken to the potential bias inherent to such kind of dataset^26,27^. In general, users of a social media may not be a random sample of the beneficiaries of a service and, the users may be biased in, for example, gender, age, and location. In our case, assessment of such bias is difficult because the attributes of the users were not public in many cases. Therefore, potential influences of such biases on our results should be kept in mind. For example, if the users of the service are biased to relatively young hikers, and hiker’s preference to vegetation is age-dependent, importance of vegetation on the recreation service quantified in this study may be under- or over-estimated.

## Conclusions

We demonstrated that associations between vegetation variables and number of hiking records as a surrogate of a recreation service flow are considerably variable. Even the signs of the associations are reversed among nearby sites depending on other variables such as accessibility and altitude. Such complexity may arise from backgrounds of beneficiaries (e.g. natural riches of their resident area) and functional responses of vegetation to a disturbance (e.g. dominance of deciduous species in a secondary forest). As a result of the variability as well as nonlinearity, expected impacts of 0.1 decreases in the vegetation variables varied over a range of ± 30% change in the service flow. Such essential heterogeneity in associations between the service and vegetation variables should be carefully reflected in landscape management.

## Methods

### Study area

We focused on hiking activity in the four main islands of Japan (Honshu, Hokkaido, Kyushu, and Shikoku) and nearby small islands connected to the main islands by a bridge (Fig. 1a). These islands lie between latitudes 31.0° and 45.5°N, and the total area is 361,000 km^2^. The islands are generally mountainous and tallest mountains in central Honshu exceed 3000 m a.s.l. (Fig. 1c). In Tokyo, mean monthly temperatures range between 5.2 °C in January and 26.4 °C in August, while they range between − 18.4 °C in January and 6.2 °C in August at the summit of the highest mountain, Mt. Fuji (3776 m a.s.l., Japan Meteorological Agency). In northern Honshu and Hokkaido, snow depth can exceed 1 m even at low elevations and high mountains are covered with snow even in southern Japan.

Vegetation excluding farmland and pasture covers 70.9% of the study area and the 93.9% is forest. Plantations of mostly evergreen conifers such as Japanese cedar (*Cryptomeria japonica*) occupy 37.6% of the vegetation area (National Surveys on the Natural Environment by the Biodiversity Center of Japan 2nd–7th; http://www.biodic.go.jp/trialSystem/top_en.html). Secondary vegetation after past human disturbances occupies 39.4% of the total vegetation and the remaining 23.0% is primary vegetation. The typical primary vegetation types are, from north to south, boreal mixed forest, deciduous broad leaved forest, and evergreen broad leaved forest.

### Grid squares

Records of hiking activity were summarized for 4244 secondary grid squares based on Standard Grid Square System, which was defined by the Minister’s Order of Administrative Management Agency in 1973. In the system, the secondary grid was defined as a grid of 5′ in latitude and 7′ 30″ in longitude, which roughly corresponds to a 10 km grid in the study area. This is the standard grid system of the government and we adopted the system for convenience in future application uses and communication with practitioners. The grids, which are defined by latitude and longitude, are different in the area up to 22% between the north and south ends. Therefore, area of each grid was included in a model as an offset term.

### Hiking activity

According to a government survey in 2016, (the Survey on Time Use and Leisure Activities by the Statistics Bureau of Japan, http://www.stat.go.jp/english/data/shakai/index.htm), 10.0% (about 10.7 million people) of Japan’s population age 15 or over enjoyed hiking/mountaineering in the last year. The census showed also that hiking is more popular among urban residents in the metropolitan areas. Both multi-day expedition to high mountains and day trek to low mountains in suburban areas are popular. Because of the severe winter climate, unskilled hikers use the high mountains in summer and early autumn only. During a summer vacation, whose peak time in Japan is August, many hikers enjoy multi-day trips to distant mountains. Spring and autumn are also popular seasons because of the mild weather and the scenic beauty of the fresh green or autumn colors.

### Data collection

In this study, we used number of hiking records accumulated on the most popular social networking service for hikers in Japan (Yamareco; https://www.yamareco.com) as a surrogate for flow of recreation service. For all the registered destinations in the study area, the number of hiking records for each month and the latitude and longitude of the destination were collected from the service in September 2016 with the *rvest*^28^ package in R software^29^. This service launched in October 2005 hosts records of the hiking route, photos, participants, and impressions of a hiking trip and facilitates communication among users. Although monthly number of records for each destination is always available on the site, the exact date of each hiking record is not always public information for privacy reasons; therefore, all of the records from the almost 11 years since the start of the service were lumped together in our analysis. Hikers may record multiple places in a single trip, so the total number of records must be larger than the number of unique trips. Users of the service sometime record a place that is not a destination, e.g. start points and stations of trails, parking areas, stations of transports, and bus stops. Such records were excluded before analyses as far as it can be judged from the name of the place. As a result, the total number of hiking records was 4,708,229 records for 16,179 destinations. Finally, these records were assigned to the 4244 grids based on the latitude and longitude of each destination and then total number of records for each grid was used as a surrogate of the recreation service flow in our analysis. Not only total number but also monthly number was used in our analysis to examine seasonal changes in associations between the service and vegetation. Total record number of the grids was strongly right-skewed; no record (handled as 0 in our analysis) was found in 2036 grids while mean and maximum record number were 1109 and 350,384, respectively.

### Explanation variables

Fifty ecological, environmental, and social/infrastructural variables (Table S1) were prepared for each grid by using ArcGIS version 10.5 (ESRI, Redlands, CA, USA). For vegetation and land-use attributes, National Surveys on the Natural Environment by the Biodiversity Center of Japan (2nd–7th; http://www.biodic.go.jp/trialSystem/top_en.html) and National Land Numerical Information (http://nlftp.mlit.go.jp/ksj-e/index.html) were used. The proportion of sea, that of total vegetation cover (excluding agricultural land and pasture) to land area, that of agricultural land (including pasture) to land area, that of natural vegetation (vegetation excluding plantations) to total vegetated area, and that of primary vegetation (vegetation with no record or evidence of a disturbance) to natural vegetation were summarized at four spatial scales: a radius of 10 km, 20 km, 50 km, and 100 km from the center of each grid. Spatial patterns of the three vegetation variables in 10 km radius were summarized in Fig. 1d–f.

Maximum elevation, minimum elevation, and ruggedness (index of topographic heterogeneity^30^) were summarized at the four spatial scales based on a digital elevation model (10-m resolution) provided by the Geospatial Information Authority of Japan (https://fgd.gsi.go.jp/download/menu.php). For climatic variables (annual and monthly mean temperature, annual and monthly precipitation, annual and monthly hours of sunshine, and annual maximum snow depth), the National Land Numeric Information provided by the Ministry of Land, Infrastructure, Transport and Tourism of Japan (http://nlftp.mlit.go.jp/ksj-e/index.html) was referenced. Densities of population and roads at the four spatial scales were prepared from population census data from the Statistics Bureau of Japan (http://e-stat.go.jp/SG2/eStatGIS/page/download.html) and the National Land Numeric Information. For calculation of these densities, the sea surface was excluded. In addition, latitude and longitude of center of each grid were also used as explanatory variables to average effects of spatial coordinates.

### Statistical analysis

In this study, we employed BRT, a machine-learning method based on regression trees^31^ for modeling the complex relationship between a CES flow and landscape attributes^12^. BRT is an ensemble learning method where multiple regression trees are sequentially combined to minimize the loss function by means of gradient descent. This technique has advantage in the development of a model with a high predictive performance, in which high-dimensional interactions among explanatory variables and nonlinear responses are fully accounted for. In ecology, BRT has been frequently used for modeling of a species distribution^32^.

Total and monthly numbers of hiking records were modeled as a function of the 50 variables described above under the assumption of a Poisson response. For temperature, precipitation, and hours of sunshine, annual and monthly average were used for the analysis of total and monthly records, respectively. In modeling by BRT, parameters for building of each learner and assembly of the learners must be carefully chosen to maximize generalization ability of a model^31^. In our case, candidate parameters were 2, 5, and 10 for the maximum depth of variable interactions for each learner; 2, 5, 10, and 20 for the minimum number of observations in the terminal nodes for each learner; 0.5 and 0.75 for the proportion of training data used for building each learner; and 1000, 2000, 4000, 6000, 8000 and 10,000 for the total number of learners (Table S2). In the model assembling process, the value of 0.01 was used as a shrinkage parameter. Ten-fold cross validation was used to obtain the best suites of parameters. R^2^ based on sum of squares:

$${R}^{2}=1-\frac{{\sum ({y}_{i}-\widehat{{y}_{i}})}^{2}}{{\sum ({y}_{i}-\overline{{y }_{i}})}^{2}}$$

was used for evaluation of the model’s prediction performance. The importance of explanatory variables was evaluated as an increase of mean absolute error after 100-times permutation of a variable^33^.

Effects of each explanatory variable (a landscape attribute) on the response variable (record number) and the context dependence were visually inspected by individual conditional expectation (ICE) plot^34^. ICE plot visualizes the effect of a given explanatory variable for each observation by connecting outcome of a model for shifting values of the focal explanatory variable throughout the range while keeping other explanatory variables as the original value. Predictions were performed in log-scale and each line was centered to be zero at the left end of the x-axis to show relative effects of explanatory variables (c-ICE plot sensu Goltstein et al.^34^). Each line in ICE plot can be colored based on value of the second explanatory variable to assist assessment of the interactive effects of the two predictors. Friedman’s H statistic^35^ was used to detect explanatory variables whose interaction with the vegetation variables are important and therefore should be used for color-coding of an ICE plot. Friedman’s H is defined as a proportion of variance of partial dependence estimates explained by interactive effects for arbitrary suites of explanatory variables.

Then, expected impacts of 0.1 decrease in the three local vegetation variables were assessed by the trained model and mapped. Although vegetation variables were sometimes more important at larger spatial scales (see “Results”), we focused on vegetation at a local (10 km radius) scale because most changes in vegetation occur at the scale in Japan (National Surveys on the Natural Environment by the Biodiversity Center of Japan, https://www.biodic.go.jp/kiso/fnd_list_h.html).

All statistical analyses were performed using the R software packag^29^. The *gbm*^36^ package was used for BRT, the *iml*^37^ package was used for calculation of Friedman’s H statistic, and the *cv.models* (Oguro, https://github.com/Marchen/cv.models) packages was used for cross validation and parameter tuning.

## Supplementary Information

Supplementary Information.

## Data Availability

All data are public and available online. Hiking records were obtained from https://www.yamareco.com. For assessment of reproducibility, we provide the original data that we collected in September 2016 as needed. Explanation variables were available at the following URLs: vegetation (http://www.biodic.go.jp/trialSystem/top_en.html), land-use (http://nlftp.mlit.go.jp/ksj-e/index.html), digital elevation model (https://fgd.gsi.go.jp/download/menu.php), climate (http://nlftp.mlit.go.jp/ksj-e/index.html), and population (http://e-stat.go.jp/SG2/eStatGIS/page/download.html).
