# Supplementary material for: Variable effects of vegetation characteristics on a recreation service depending on natural and social environment
Source: Sci Rep. 2023 Jan 13;13:684. doi: 10.1038/s41598-023-27799-7 (PMC9839729; doi:10.1038/s41598-023-27799-7)
Supplement: Supplementary file 1 — Supplementary Information. [file 41598_2023_27799_MOESM1_ESM.pdf]

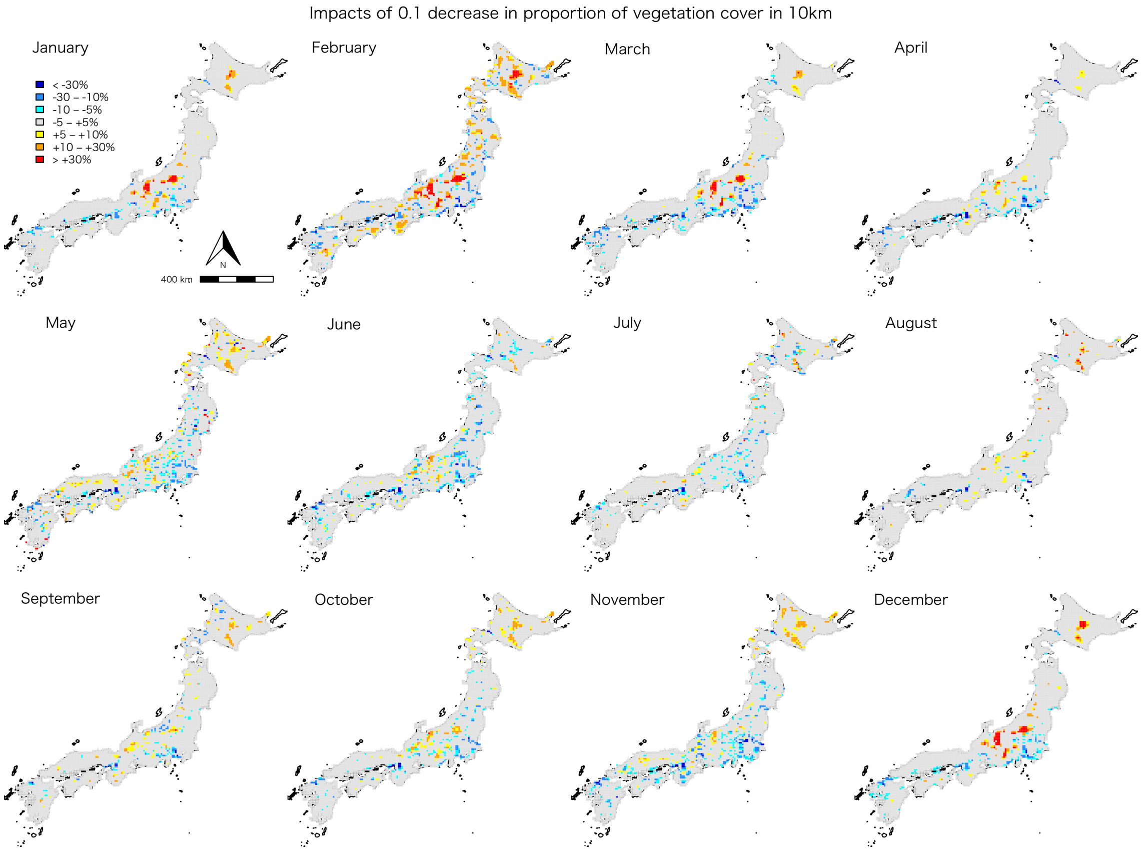

Fig. S1

Expected impacts of 0.1 decrease in proportion of vegetation cover on number of hiking records for each month.

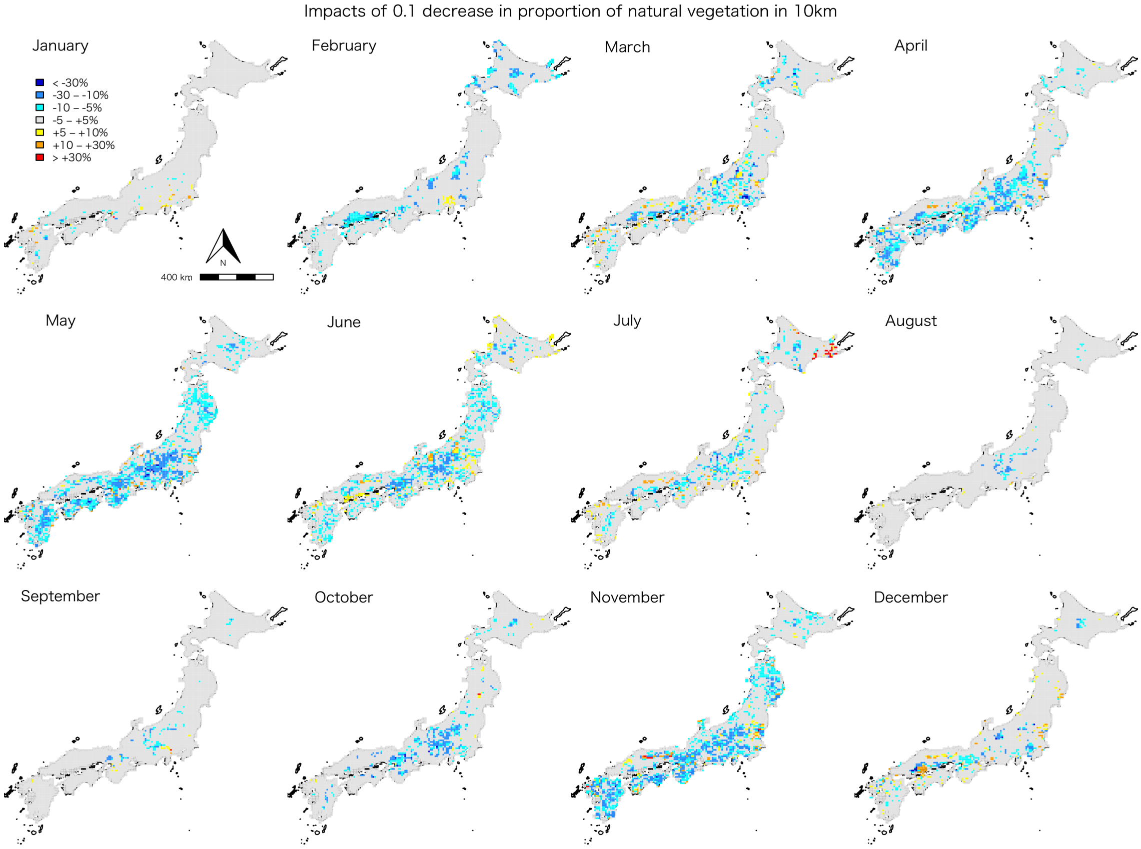

Fig. S2

Expected impacts of 0.1 decrease in proportion of natural vegetation to total vegetation cover on number of hiking records for each month.

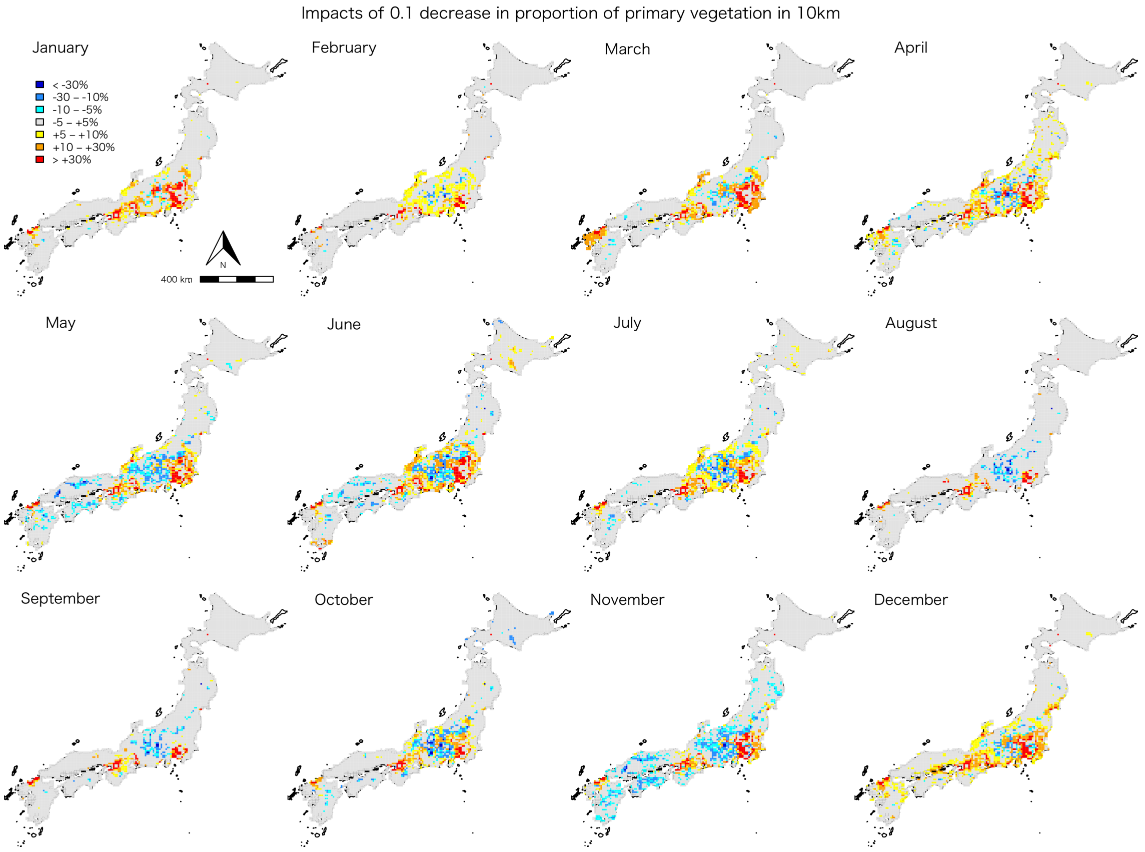

Fig. S3

Expected impacts of 0.1 decrease in proportion of primary vegetation to natural vegetation on number of hiking records for each month.

Explanation variables	Unit
Longitude	°
Latitude	°
Annual (or monthly) mean temperature	°C
Annual (or monthly) rainfall	mm
Annual (or monthly) sunshine	hours
Maximum snowdepth	cm
Population density in 10, 20, 50, and 100 km radius	person km^{-2}
Road density in 10, 20, 50, and 100 km radius	km km^{-2}
Cultural tourism resources in 10, 20, 50, and 100 km radius	sites km^{-2}
Maximum altitude in 10, 20, 50, and 100 km radius	m
Minimum altitude in 10, 20, 50, and 100 km radius	m
Topographic heterogeneity in 10, 20, 50, and 100 km radius	-
Proportion of sea surface in 10, 20, 50, and 100 km radius	km^2 km^{-2}
Vegetation cover in 10, 20, 50, and 100 km radius	km^2 km^{-2}
Proportion of natural vegetation in 10, 20, 50, and 100 km radius	km^2 km^{-2}
Proportion of primary vegetation in 10, 20, 50, and 100 km radius	km^2 km^{-2}

Table S1. List of the 50 explanatory variables

Maximum depth of variable interactions for each learner	2, 5, 10
Minimum number of observations in the terminal nodes for each learner	2, 5, 10, 20
Proportion of training data used for building each learner	0.5, 0.75
Total number of learners	1000, 2000, 4000, 6000, 8000, 10000
Shrinkage	0.1

Table S2 Summary of the hyperparameters of gradient boosting
